# The Relationship between Dietary Flavonols Intake and Metabolic Syndrome in Polish Adults

**DOI:** 10.3390/nu15040854

**Published:** 2023-02-08

**Authors:** Joanna Popiolek-Kalisz

**Affiliations:** 1 Clinical Dietetics Unit, Department of Bioanalytics, Medical University of Lublin, ul. Chodzki 7, 20-093 Lublin, Poland; joanna.popiolek-kalisz@umlub.pl; 2 Department of Cardiology, Cardinal Wyszynski Hospital in Lublin, al. Krasnicka 100, 20-718 Lublin, Poland; 3 Department of Biotechnology, Microbiology and Human Nutrition, University of Life Sciences in Lublin, ul. Skromna 8, 20-704 Lublin, Poland

**Keywords:** flavonols, metabolic syndrome, quercetin, dyslipidemia, kaempferol, isorhamnetin

## Abstract

Metabolic syndrome (MetS) is a cluster of metabolic disorders primarily caused by central obesity, which results in chronic inflammation leading to hypertension, diabetes and atherogenic dyslipidemia. Inflammation underlying MetS could be the target for dietary flavonols as they present antioxidative properties. The aim of this paper was to analyze the differences in habitual intake of selected flavonols (quercetin, kaempferol, isorhamnetin and myricetin) between MetS patients and healthy participants, and its relationship with MetS advancement. Ninety participants were enrolled in this study. The one-year flavonol intake was assessed with a dedicated food frequency questionnaire. The patients with MetS consumed significantly less quercetin (*p* = 0.01), kaempferol (*p* = 0.04), isorhamnetin (*p* < 0.001), total flavonols (*p* = 0.01), tomatoes (*p =* 0.001) and wine (*p* = 0.01) daily. Further analysis revealed a moderate inverse correlation between quercetin (*p* = 0.001), kaempferol (*p* = 0.01), isorhamnetin (*p* < 0.001), total flavonols (*p* = 0.001) and tomato consumption (*p* = 0.004) and MetS stage. The analysis of laboratory parameters showed that dietary intake of flavonols was not correlated with lipid profile, glucose level or renal function. On the basis of this observation, a potential protective effect of dietary flavonols, mainly from tomatoes, against MetS could be suggested. However, when referring to MetS components, flavonols probably mainly impact central obesity and blood pressure, without a significant impact on conventional lipid-profile parameters and glucose level.

## 1. Introduction

Metabolic syndrome (MetS) is becoming a global problem that is associated with the progressive change in the lifestyle of modern societies. MetS is a cluster of metabolic disorders mainly caused by central obesity [1]. They include insulin resistance, atherogenic dyslipidemia, central obesity and elevated blood pressure.

MetS has become an important healthcare problem. It is estimated that MetS prevalence in the US reached 35% in 2012, which was a 10% increase compared to a 25% prevalence in 1994 [2]. The situation in Europe is better, but still very serious, as MetS prevalence was about 24% in 2015 [3]. The problem is very important, because MetS leads to an increased risk of diabetes (if not already present) and cardiovascular disease (CVD). CVD and diabetes are the leading causes of death globally [4].

MetS pathogenesis is very complex; however, it generally initiates from excess visceral fat tissue, which via adipokine production leads to insulin resistance, chronic low-grade inflammation and neurohormonal activation. Secondarily, these processes result in endothelium dysfunction and glucose and lipid metabolism abnormalities, among other problems. The fat tissue distribution is an important factor as visceral adipose tissue is more vulnerable to macrophage infiltration and thus to inflammation development [5] and free fatty acid release [6].

According to the new IDF definition, MetS diagnosis requires the presence of central obesity, which is defined as a waist circumference of 94 cm or more in men or 80 cm or more in women in Poland, or a BMI of 30 kg/m^2^ or higher, accompanied by two out of four additional criteria: (1) elevated blood triglycerides (TG) 150 mg/dL or greater (or hypertriglyceridemia treatment), (2) reduced high-density lipoprotein cholesterol (HDL) less than 40 mg/dL in men or less than 50 mg/dL in women (or hypercholesterolemia treatment), (3) elevated fasting glucose of 100 mg/dL or greater (or diabetes treatment), (4) blood pressure values of systolic (SBP) 130 mmHg or higher and/or diastolic (DBP) 85 mmHg or higher (or hypertension treatment) [7]. Although insulin resistance is the main mechanism of MetS, it is not directly captured in the diagnostic criteria as insulin level measurement (essential for this purpose) is cumbersome in everyday clinical practice. However, insulin resistance is represented by the waist circumference criterion, because they correlate [8].

Excessive fat tissue located in the abdomen area, which is the foundation of MetS, is the result of interactions among lifestyle factors and genetic predispositions [9]. The main lifestyle contributors include improper dietary patterns (mainly high caloric intake) and lack of physical activity.

In connection with the mechanisms described above, MetS prevention and treatment are based on lifestyle changes, including dietary modifications. They involve, e.g., increased consumption of fruit and vegetables, which are good sources of antioxidants and fiber. There have not been established any recommendations for antioxidative agent supplementation or details of their dietary intake in terms of MetS prevention [10]. Flavonols are a group of flavonoids distinguished by their chemical structure, including a 3-hydroxyflavone backbone, which are known for their antioxidative properties. They are present mainly in fruits, vegetables, and tea. The most important flavonols are quercetin and kaempferol, followed by less-prevalent compounds such as myricetin, isorhamnetin, morin, galangin, fisetin, kaempferide, azaleatin, natsudaidain, pachypodol and rhamnazin. The major contributors to everyday dietary flavonol intake are onions, tea, and apples [11]. Other flavonol-rich products are kale, lettuce, tomatoes, broccoli, grapes, berries, and red wine [12,13]. Studies of CVD patients showed that the main dietary contributors to flavonol intake are blueberries and apples among the fruits; onions and tomatoes among the vegetables; and tea (black and green), coffee and wine among the beverages [14].

Low-grade inflammation underlying MetS could be the target for flavonols as they present antioxidative properties. Existing findings suggest a positive impact of selected flavonols, mainly quercetin, on MetS single components; however, there are not many studies investigating the impact of single flavonols intake on MetS as a set of disorders. The results of the already-conducted studies are not consistent. What is more, most of the studies focus only on quercetin, while there is a shortage of studies investigating other flavonols’ impact on metabolic parameters. There has not been any study conducted summarizing all the components of MetS together.

The objective of this study was to analyze the differences in habitual dietary intake of flavonols (quercetin, kaempferol, isorhamnetin and myricetin) between patients with and without MetS and the relationship between their habitual intake and MetS advancement. Additionally, the relationship between dietary flavonols intake, their main dietary sources consumption and metabolic parameters (glucose level and lipid profile) in MetS patients was also investigated. The main research question was: Is there a relationship between flavonols intake and MetS? The hypotheses were: (i) Does flavonols intake differ between MetS and healthy participants? (ii) Is flavonols intake related to MetS advancement? (iii) Is flavonols intake related to the main laboratory parameters in MetS patients?

This is the first study that addresses the intake of all four main flavonols, not only quercetin, on MetS. Moreover, in the course of this study, the relationship between dietary flavonols intake and MetS as a holistic set of disorders was analyzed. As explained above, the components of MetS are usually related to each other; thus, analysis of only single ones separated from others could potentially not provide all the information. The foregoing studies were focused on single components of MetS, but there has not been any analysis conducted which acknowledged MetS as a set of disorders together. This is the first study to present such an attitude.

## 2. Materials and Methods

Ninety participants (53 women and 37 men) were enrolled in this study between April and December 2022. The inclusion criteria were: (1) age 18–85 years, (2) written consent and (3) mental condition that enabled a one-year retrospective dietary interview. The exclusion criteria were: (1) age <18 or >85 years, (2) lack of written consent, (3) abnormal mental condition, (4) pregnancy and (5) special diet due to health reasons.

The food-frequency questionnaire dedicated to one-year specific flavonol intake assessment was administered to the participants [15]. The questionnaire gathered information about the mean consumption of 140 flavonol sources during the preceding year. The full questionnaire is available as supplementary material [15]. The selected flavonols were the four most widespread in food sources according to the USDA database [16]. The suggested portions of the products were based on typical servings in everyday life (e.g., one piece, a glass) and described for the participants by a suggested serving (e.g., a piece, a glass) and a weight in grams. The participants were asked to provide a frequency of selected product consumption (never or almost never, once a month, few times a month with a number of times per month given by the responder, once a week, few times a week with a number of times per week given by the responder, once a day, few times every day with a number of times per day given by the responder). The amounts of quercetin, kaempferol, isorhamnetin, and myricetin in each product were based on the data available in the USDA database [16]. On the basis of this information, the mean daily consumption of each product and flavonol was calculated for each participant. Total flavonol intake was calculated by adding the values of quercetin, kaempferol, isorhamnetin, and myricetin. The daily intake of each compound was expressed relative to body mass. The patient’s weight was measured with 0.05 kg accuracy by a trained professional. The patient was permitted to wear only underwear for this measurement. The information about the mean daily intake of flavonol sources was also derived from the above-described questionnaire [15].

The fasting glucose, lipid profile and creatinine level were assessed in venous blood. The patients were not allowed to eat for 12 h before the test. The blood samples were gathered by a trained nurse. The samples for glucose tests were gathered with dedicated probes (EDTA + sodium fluoride) and then measured using the enzyme (hexokinase) method with a Cobas Pro (Roche Diagnostics, Mannheim, Germany) analyzer. The lipid profile test samples were gathered with dedicated probes (heparinized) and then performed using colorimetric enzyme assays with a Cobas Pro (Roche Diagnostics, Mannheim, Germany) analyzer. Creatinine samples were gathered with dedicated probes (heparinized) and then measured using a colorimetric test based on the Jaffe method with a Cobas Pro (Roche Diagnostics, Mannheim, Germany) analyzer.

### 2.1. Ethical Concerns

The study was approved by the local Bioethics Committee of the Medical University of Lublin (consent no. KE-0254/9/01/2022). The study was conducted in line with the directives of the Declaration of Helsinki on Ethical Principles for Medical Research. All participants signed a written consent agreement.

### 2.2. Statistical Analysis

Statistical analyses were performed with the RStudio software v. 4.2.0. The normality of the distribution of each parameter was checked with the Shapiro–Wilk test. The variables were presented as means (±SD). The MetS diagnosis was based on fulfilling the above-mentioned criteria (1 basic and 2 additional criteria). The comparison of flavonol intake between patients with and without MetS was performed with Mann–Whitney tests. A *p* value below 0.05 was considered significant. Then, the MetS stage was expressed as the number of fulfilled criteria of MetS. The one-way ANOVA test was used to compare the mean flavonol intakes between these groups. Then the linear correlation between flavonol intake and MetS progression was investigated with the Pearson correlation test. The cut-off points used for the correlation coefficient were the same as above: <0.20 as low, 0.20–0.49 as moderate and ≥0.50 as high correlation. A *p* value below 0.05 was considered significant. The two-way ANOVA was used to compare the mean flavonol intakes between the subgroups regarding BMI impact. A *p* value below 0.05 was considered significant.

Pearson correlation was also used to analyze the linear association between selected flavonol mean daily intake and laboratory parameters, and between selected product mean daily intake and laboratory parameters. The cut-off points used for the correlation coefficient were as follows: <0.20 as low, 0.20–0.49 as moderate and ≥0.50 as high correlation. A *p* value below 0.05 was considered significant.

## 3. Results

### 3.1. The Characteristics of the Participant Group

The final group included 89 Europid patients (55 women and 37 men). One participant with MetS was excluded from the analysis due to blood sample hemolysis. A total of 32 participants met the MetS diagnosis criteria and 57 participants were not diagnosed with MetS.

The mean age was 45.8 ± 21.9 years. The patients were non-smokers. The mean daily intakes for each flavonol were as follows: 0.63 ± 0.39 mg/kg for quercetin, 0.22 ± 0.13 mg/kg for kaempferol, 0.06 ± 0.06 for isorhamnetin, 0.08 ± 0.05 for myricetin and 0.98 ± 0.57 mg/kg for total flavonols. The mean body mass was 71.39 ± 14.49 kg and the mean BMI was 25.34 ± 4.98 kg/m^2^.

The MetS group was older (67.29 ± 9.12 years) than healthy controls (33.56 ± 17.35 years) and included a smaller percentage of women (46%) than in healthy controls (67%).

### 3.2. Flavonols Intake in Participants with and without MetS

The subgroup analysis between the patients diagnosed with MetS and those without MetS revealed significant differences in total flavonol (*p* = 0.001), quercetin (*p* = 0.01), kaempferol (*p* = 0.04) and isorhamnetin (*p* < 0.001) intakes.

**Figure 1:**
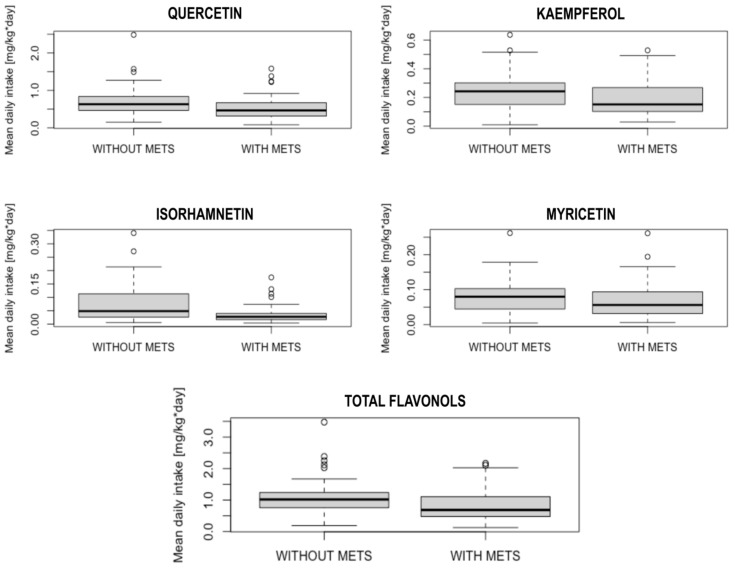
Box-plots presenting the differences in flavonols intake between patients with and without MetS diagnosis. (MetS—metabolic syndrome).

Among flavonol sources, patients without MetS eat significantly more tomatoes than patients with MetS (0.94 ± 0.76 portions/day vs. 0.58 ± 0.72 portions/day, *p* = 0.001) and drink more wine (0.15 ± 0.32 portions/day vs. 0.08 ± 0.21 portions/day, *p* = 0.01). The detailed results are presented in Table 1. The box-plots showing the differences in flavonols intake between patients with and without MetS diagnosis are presented in Figure 1.

**Figure 2:**
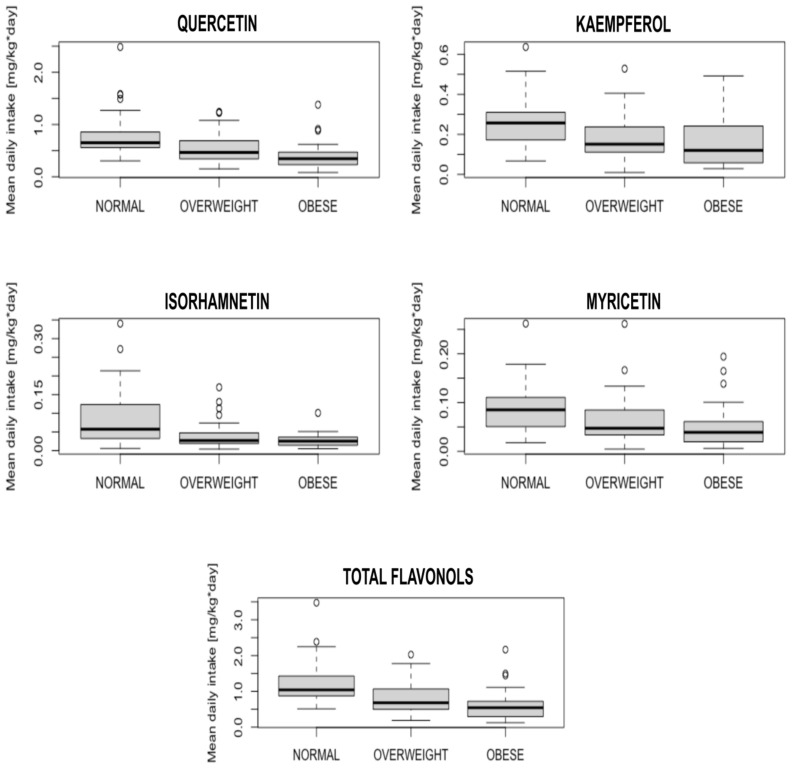
Box-plots presenting the differences in flavonols intake between subgroups with normal body mass, overweight and obese.

When participants were divided into subgroups by BMI—normal (<25kg/m^2^), overweight (25–29.99 kg/m^2^), and obese (≥30 kg/m^2^)—significant differences were present for all flavonols: quercetin (*p* < 0.001), kaempferol (*p* = 0.004), isorhamnetin (*p* = 0.001), myricetin (*p* = 0.01) and total flavonols (*p* < 0.001). The detailed results are presented in the box-plot in Figure 2.

### 3.3. Flavonols Intake and MetS Advancement

The comparison of flavonol intake between the subgroups meeting consecutive numbers of MetS criteria showed that total flavonol (*p* = 0.003), quercetin (*p* = 0.005), kaempferol (*p* = 0.03) and isorhamnetin (*p* < 0.001) intakes differed significantly between these subgroups. The detailed results are presented in Table 2.

The further analysis revealed linear characteristics of these relationships (R: −0.31; 95% CI: −0.486 to −0.108; *p* = 0.003 for total flavonols; R: −0.30; 95% CI: −0.476 to −0.095; *p* = 0.001 for quercetin; R: −0.23; 95% CI: −0.421 to −0.026; *p* = 0.01 for kaempferol; R: −0.40; 95% CI: −0.559 to −0.206; *p* < 0.001 for isorhamnetin). Among flavonol sources, MetS advancement was inversely correlated with tomato consumption (R: −0.30, 95% CI: −0.483 to −0.103; *p* = 0.004). Detailed results are presented in Table 3.

The analysis regarding flavonol intakes and BMI impact showed significant differences between subgroups in terms of each flavonol: quercetin, kaempferol, isorhamnetin, myricetin and total flavonols (*p* < 0.001 for each). When participants were divided into subgroups by BMI—normal (< 25kg/m^2^), overweight (25–29.99 kg/m^2^) and obese (≥ 30 kg/m^2^)—the correlation was present only in the overweight subgroup for isorhamnetin intake (R: −0.38; 95% CI: −0.623 to −0.012; *p* = 0.04) and tomato consumption (R:−0.47; 95% CI: −0.712 to −0.122; *p* = 0.01).

### 3.4. The Analysis of Laboratory Parameters in MetS Patients

The analysis of the relationship between flavonol intake and laboratory metabolic parameters (glucose, TC, HDL, LDL, TG, creatinine) revealed that total and selected flavonol intake was not correlated with any of them. The detailed results are presented in Table 4. The subgroup analysis did not show any significant correlation in men and women or in BMI-stratified subgroups. Mean kaempferol intake was highly inversely correlated with glucose level among men; however, this relationship was still not significant (R: −0.58; 95% CI: −0.876 to 0.027; *p* = 0.06).

### 3.5. The Analysis of the Flavonol Source Consumption in MetS Patients

The results showed that onion and tomato were the main contributors to flavonol intake among vegetables, blueberries and apples among fruit, tea and coffee among non-alcoholic beverages and wine as an alcoholic drink. The analysis of the relationship between these flavonol sources’ intake and laboratory metabolic parameters (glucose, TC, HDL, LDL, TG, creatinine) did not reveal any significant correlation. The detailed results are presented in Table 5. The subgroup analysis in MetS patients regarding BMI (overweight and obese) showed a significant correlation in the overweight subgroup for tomato consumption and TC (R = −0.68; 95% CI: −897 to −0.214; *p* = 0.01), TG (R = −0.64; 95% CI: −0.882 to −0.144; *p* = 0.02) and LDL levels (R = −0.58; 95% CI: −0.858 to −0.047; *p* = 0.04).

## 4. Discussion

MetS is the set of disorders such as central obesity, elevated blood pressure, elevated glucose level and atherogenic dyslipidemia which is defined as elevated TG and decreased HDL levels. All of them are the main risk factors of CVD. This is why MetS prevention and treatment are crucial for CVD prevention as well.

This study is the first to analyze MetS as a set of disorders, not only separate conditions, in terms of dietary flavonol intake. The analysis of the flavonols intake between patients with and without diagnosed MetS revealed significant differences in quercetin, kaempferol, isorhamnetin and total flavonol consumption. The MetS patients were characterized by lower quercetin, kaempferol, isorhamnetin and total flavonol intakes compared to healthy ones. Thus, a preliminary conclusion can be provided that dietary flavonol consumption could play a potentially protective role against MetS development. Then, to analyze the relationship between flavonols intake and MetS progression, a comparison between the subgroups meeting the consecutive numbers of MetS criteria was performed. There are no defined stages of MetS as its criteria are complex, which is why this simplified form of MetS advancement was used for the purpose of this study. This detailed investigation revealed significant differences in quercetin, kaempferol, isorhamnetin and total flavonol intake also between the subgroups meeting different numbers of MetS criteria. Further analysis showed the linear nature of this relationship, as quercetin, kaempferol, isorhamnetin and total flavonol habitual intake were moderately inversely correlated with MetS stage. The details of this trend were the subject of further investigation in the course of this study.

Among flavonols sources, there were significant differences in tomato and wine consumption between patients with and without MetS. Tomatoes are good sources of flavonols, mainly kaempferol and quercetin (from 15 μg/mL in juice to 70 μg/g in puree) [17,18]. It is worth noting that they also contain other bioactive ingredients such as lycopene, which is also proven to present a protective potential against MetS [19]. Apart from the mentioned study focusing on lycopene from tomatoes, no other human study is available that links tomato consumption with MetS incidence. It is also worth noting that antioxidant bioavailability in different foods could be impacted by cultivation practice, meal preparation techniques or storage. Most of the phenolic compounds are located in tomato skin [17]. Quercetin and kaempferol content in tomatoes decreases in the course of peeling, dicing and heat treatment [20,21]. On the other hand, studies show that lycopene bioavailability from tomatoes increases after thermal treatment [22]. Storage of tomato-derived industry-processed products did not change the content of quercetin [23].

In the present study, wine consumption is inversely associated with MetS occurrence, which is in line with other observational studies regarding moderate wine consumption [24,25]. The mechanisms of this phenomenon could include wine polyphenols’ impact on the gut microbiota [26]. On the other hand, the direct positive impact of wine polyphenols on MetS laboratory components was not confirmed [27], which is also in line with the present study. Nonetheless, it is worth noting that wine consumption could be part of a more complex lifestyle pattern that could play an additional role in MetS prevention [28]. What is more, heat treatment increases flavonol content in wine pomace [29].

As already mentioned, MetS is a set of disorders. Referring to BMI values, significant differences were observed regarding all flavonol intake between participants with normal body mass, overweight and obese. Central obesity prevalence, which is the primary cause of MetS, has already been linked to lower flavonol consumption [30]. Excessive fat adipose tissue presence, especially in the abdominal region, leads to chronic inflammation, which is one of the causes of insulin resistance and endothelium dysfunction. They result in other MetS components: hypertension, diabetes and atherogenic dyslipidemia development. A positive impact of quercetin supplementation on blood pressure levels has been observed [31,32]. As one of the MetS criteria is elevated blood pressure level, this group of patients could possibly benefit from quercetin supplementation [33,34]. The relationship between habitual flavonol consumption and blood pressure in men was also confirmed in observational studies [14]. This is why these parameters were not the subject of investigation in the course of this study.

In terms of glucose metabolism, this study showed that flavonol consumption was not correlated with the fasting glucose level. On the other hand, interventional studies in diabetic patients showed that supplementation of quercetin and myricetin could decrease glucose levels [35,36,37]. The difference might have been caused by the fact that none of these studies was conducted in MetS patients, i.e., with other comorbidities apart from diabetes. Mean kaempferol intake was highly inversely correlated with glucose level among men; however, this relationship was still not significant. There is not any data available from human studies that investigated this particular topic. Other parameters related to glucose metabolism, such as insulin or glycated hemoglobin, were not analyzed because they are not acknowledged in the MetS criteria at the moment.

Flavonol intake was not correlated with lipid parameters as well. This observation is in line with results by other authors, as among MetS patients, quercetin supplementation did not alter total cholesterol (TC), triglycerides and the LDL/HDL, TC/HDL and triglycerides/HDL ratio [33]. Nonetheless, in the same study, it significantly decreased plasma concentrations of oxidized LDL, which is known for its atherogenic influence [33]. In the present study, only basic lipid parameters available in everyday clinical practice were measured; however, more detailed observation regarding other parameters such as oxidized LDL would be helpful. In interventional studies, the combination of quercetin and kaempferol also did not improve the lipid profile in healthy men; however, the applied doses of 20.2 mg and 3.4 mg, respectively, were relatively low [38] compared even to habitual intake, which was 40.0 mg/day for quercetin and 14.0 mg/day for kaempferol in the present study. What is more, the participants of this study were non-smokers, while the significant positive impact of quercetin supplementation in other studies was observed only in smokers [35].

Renal function is an essential aspect of the clinical assessment of MetS patients; however, it is not acknowledged as a MetS criterion. The present analysis showed that flavonol consumption was also not correlated with creatinine levels. The results from animal model studies suggest that quercetin has a protective role against diabetic nephropathy, as well as in human clinical trials [39,40]. The differences might be caused by the quercetin dosage, as in the interventional environment high daily doses, such as 500 mg and 1000 mg, were applied, while the observed mean habitual intake was 41.3 mg/day.

Among other flavonol sources, tomato intake was moderately inversely correlated with TC and LDL levels; however, this relationship was not significant. Nonetheless, tomato consumption was highly inversely correlated with TC, TG and LDL levels in the overweight subgroup. This could suggest a potential beneficial role of tomato consumption in overweight MetS patients. This observation is in line with the studies suggesting a beneficial role of tomato juice supplementation in MetS [41]. In the mentioned study, tomato juice supplementation did not significantly decrease TC levels; however, it significantly decreased LDL levels and elevated HDL levels. The differences might have been caused by different dosages; however, it is unclear as the authors described that the patients were told to consume tomato juice four times per week, but they did not detail the portion capacity to evaluate the mean daily flavonoid intake.

This study has its limitations. The flavonol intake was based on questionnaire responses, so it shares all the limitations of this type of study. The cross-sectional type of the study does not allow the establishment of a cause/result relationship, so these observations should be confirmed in an interventional study. Even though the questionnaire used was proved suitable for this type of study and the study aimed to investigate long-term habits, additional blood tests for flavonol serum levels could support these observations. Moreover, the questionnaire was based on the USDA database; thus, it could not analyze the year-long consumption of each possible variety of every available fruit or vegetable. What is more, laboratory data were available only in MetS patients, so future laboratory analysis including healthy participants would be helpful. The healthy controls were volunteers, and women were more interested in study participation; thus the majority of the group was women. Moreover, healthy participants showed more interest in the study participation; thus, the sizes of the MetS and control groups were not equal, which disabled age or sex matching. Nonetheless, this is the first study to analyze the relationship between dietary flavonols as single compounds and the complexity of MetS. This approach is highly valuable, as it provides a direction for future interventional studies regarding the preventive role of selected flavonols in MetS. This study aimed to analyze general clinical trends; however, a detailed investigation of the mechanisms responsible for these observations is needed, as it might be beyond the antioxidative properties.

## 5. Conclusions

This study showed that healthy participants consume more flavonols than those with MetS. Moreover, higher habitual flavonol intake was inversely associated with MetS progression. On the basis of this observation, a potential protective effect of dietary flavonol intake against MetS could be suggested. However, when referring to MetS components, habitual intake of selected flavonols was related mainly to central obesity and blood pressure, without a significant correlation with conventional lipid profile parameters or glucose levels. Further investigation regarding additional parameters of lipid and glucose metabolism could provide additional information upon this topic. Among flavonols sources, the patients without MetS ate significantly more tomatoes than MetS patients, which consumption was also inversely correlated with MetS stage. This could suggest a potential role of tomato consumption in MetS prevention.

## Figures and Tables

**Table 1:** The comparison of mean daily flavonols intake and flavonol source consumption between the patients with and without metabolic syndrome.

Flavonol Intake [mg/kg × Day]	Without MetS (n = 57)	SD	With MetS (n = 32)	SD	*p*
Total flavonols	1.10	±0.56	0.77	±0.53	0.01
Quercetin	0.71	±0.38	0.50	±0.36	0.01
Kaempferol	0.24	±0.12	0.17	±0.12	0.04
Isorhamnetin	0.07	±0.07	0.03	±0.03	<0.001
Myricetin	0.08	±0.05	0.07	±0.06	0.19
Flavonols source consumption [portion/day]					
Onion	0.47	±0.46	0.35	±0.39	0.36
Tomato	0.94	±0.76	0.58	±0.72	0.001
Blueberry	0.31	±0.51	0.13	±0.20	0.45
Apple	0.56	±0.52	0.67	±0.50	0.19
Tea	1.80	±1.60	1.96	±1.80	0.50
Coffee	0.89	±0.96	0.67	±0.76	0.33
Wine	0.15	±0.32	0.08	±0.21	0.01

**Table 2:** The comparison of flavonol intake between the subgroups meeting the consecutive numbers of metabolic syndrome (MetS) criteria.

Mean Daily Intake [mg/kg]	0 Criteria	SD	1 Criterion	SD	2 Criteria	SD	3 Criteria	SD	4 Criteria	SD	5 Criteria	SD	*p*
Total flavonols	1.12	±0.60	1.36	±0.60	0.93	±0.30	0.89	±0.64	0.66	±0.37	0.51	±0.08	0.003
Quercetin	0.72	±0.41	0.87	±0.38	0.59	±0.19	0.59	±0.44	0.36	±0.41	0.32	±0.09	0.005
Kaempferol	0.24	±0.13	0.31	±0.21	0.22	±0.08	0.17	±0.13	0.15	±0.18	0.13	±0.02	0.03
Isorhamnetin	0.08	±0.07	0.08	±0.07	0.04	±0.01	0.04	±0.04	0.02	±0.01	0.02	±0.02	<0.001
Myricetin	0.08	±0.05	0.09	±0.03	0.07	±0.05	0.09	±0.07	0.05	±0.04	0.04	±0.01	0.16

MetS criteria: (I) central obesity (waist circumference 94 cm or more in men and 80 cm or more in women, or BMI 30 kg/m^2^ or higher); (II) elevated blood triglycerides (TG) 150 mg/dL or greater (or hypertriglyceridemia treatment); (III) reduced high-density lipoprotein cholesterol (HDL) less than 40 mg/dL in men or less than 50 mg/dL in women; (IV) elevated fasting glucose of 100 mg/dL or greater; (V) blood pressure values of systolic (SBP) 130 mmHg or higher and/or diastolic (DBP) 85 mmHg or higher.

**Table 3:** The correlation between mean daily flavonols intake and MetS stage.

	Metabolic Syndrome Stage [Fulfilled Criteria]		
Mean Daily Intake	R	95% CI	*p*
Total flavonols [mg/kg × day]	−0.31	−0.486 −0.108	0.003
Quercetin [mg/kg × day]	−0.30	−0.476 −0.095	0.005
Kaempferol [mg/kg × day]	−0.23	−0.421 −0.026	0.03
Isorhamnetin [mg/kg × day]	−0.40	−0.559 −0.206	<0.001
Myricetin [mg/kg × day]	−0.15	−0.349 −0.059	0.16
Onion [portions/day]	−0.16	−0.360 0.048	0.13
Tomato [portions/day]	−0.30	−0.483 −0.103	0.004
Blueberry [portions/day]	−0.19	−0.381 0.022	0.08
Apple [portions/day]	0.11	−0.097 0.314	0.29
Tea [portions/day]	0.11	−0.104 0.308	0.32
Coffee [portions/day]	−0.11	−0.312 0.100	0.30
Wine [portions/day]	−0.19	−0.386 0.015	0.07

**Table 4:** The correlation between mean daily flavonol intake and laboratory parameters.

Quercetin			
Parameter	R	95% CI	*p*
Glucose [mg/dL]	−0.13	−0.502 0.275	0.52
Creatinine [mg/dL]	−0.15	−0.475 0.209	0.41
TC [mg/dL]	0.10	−0.270 0.444	0.60
TG [mg/dL]	0.15	−0.219 0.486	0.42
LDL [mg/dL]	0.10	−0.270 0.444	0.60
HDL [mg/dL]	−0.20	−0.521 0.174	0.29
Kaempferol			
	R	95% CI	*p*
Glucose [mg/dL]	−0.19	−0.546 0.219	0.36
Creatinine [mg/dL]	0.03	−0.326 0.371	0.89
TC [mg/dL]	0.16	−0.214 0.490	0.40
TG [mg/dL]	0.28	−0.094 0.578	0.14
LDL [mg/dL]	0.12	−0.265 0.448	0.58
HDL [mg/dL]	−0.13	−0.466 0.244	0.50
Isorhamnetin			
	R	95% CI	*p*
Glucose [mg/dL]	−0.06	−0.445 0.342	0.77
Creatinine [mg/dL]	−0.22	−0.527 0.141	0.23
TC [mg/dL]	0.06	−0.305 0.413	0.75
TG [mg/dL]	0.07	−0.299 0.419	0.72
LDL [mg/dL]	0.02	−0.342 0.378	0.91
HDL [mg/dL]	−0.06	−0.414 0.304	0.74
Myricetin			
	R	95% CI	*p*
Glucose [mg/dL]	−0.13	−0.501 0.277	0.53
Creatinine [mg/dL]	−0.15	−0.476 0.207	0.40
TC [mg/dL]	0.07	−0.300 0.417	0.72
TG [mg/dL]	0.09	−0.281 0.435	0.64
LDL [mg/dL]	0.08	−0.285 0.431	0.66
HDL [mg/dL]	−0.10	−0.444 0.270	0.60
Total flavonols			
	R	95% CI	*p*
Glucose [mg/dL]	−0.15	−0.516 0.259	0.47
Creatinine [mg/dL]	−0.13	−0.455 0.232	0.49
TC [mg/dL]	0.11	−0.256 0.456	0.55
TG [mg/dL]	0.18	−0.193 0.507	0.34
LDL [mg/dL]	0.10	−0.268 0.446	0.59
HDL [mg/dL]	−0.18	−0.506 0.195	0.35

(TC—total cholesterol, LDL—low-density lipoprotein cholesterol, HDL—high-density lipoprotein cholesterol, TG—triglycerides).

**Table 5:** Correlation between mean daily consumption of major flavonol sources and laboratory parameters in MetS patients.

Onion			
Parameter	R	95% CI	*p*
Glucose [mg/dL]	0.06	−0.448 0.339	0.76
Creatinine [mg/dL]	−0.17	−0.492 0.187	0.34
TC [mg/dL]	0.05	−0.312 0.407	0.78
TG [mg/dL]	0.08	−0.287 0.430	0.67
LDL [mg/dL]	−0.01	−0.365 0.356	0.98
HDL [mg/dL]	−0.04	−0.393 0.326	0.84
Tomato			
Glucose [mg/dL]	−0.11	−0.482 0.300	0.61
Creatinine [mg/dL]	0.18	−0.176 0.501	0.31
TC [mg/dL]	−0.34	−0.622 0.026	0.07
TG [mg/dL]	−0.14	−0.472 0.237	0.48
LDL [mg/dL]	−0.31	−0.606 0.056	0.09
HDL [mg/dL]	−0.10	−0.441 0.273	0.61
Blueberry			
Glucose [mg/dL]	−0.04	−0.432 0.357	0.83
Creatinine [mg/dL]	−0.11	−0.438 0.252	0.57
TC [mg/dL]	−0.10	−0.443 0.272	0.61
TG [mg/dL]	0.01	−0.354 0.367	0.97
LDL [mg/dL]	−0.12	−0.460 0.252	0.53
HDL [mg/dL]	−0.03	−0.383 0.337	0.74
Apple			
Glucose [mg/dL]	−0.26	−0.591 0.155	0.22
Creatinine [mg/dL]	0.06	−0.296 0.400	0.75
TC [mg/dL]	−0.01	−0.367 0.354	0.97
TG [mg/dL]	0.01	−0.351 0.370	0.95
LDL [mg/dL]	−0.03	−0.387 0.333	0.87
HDL [mg/dL]	−0.27	−0.500 0.203	0.37
Tea			
Glucose [mg/dL]	−0.15	−0.517 0.257	0.46
Creatinine [mg/dL]	0.22	−0.136 0.531	0.22
TC [mg/dL]	0.33	−0.04 0.616	0.08
TG [mg/dL]	0.28	−0.084 0.585	0.13
LDL [mg/dL]	0.23	−0.145 0.543	0.23
HDL [mg/dL]	−0.01	−0.366 0.355	0.97
Coffee			
Glucose [mg/dL]	−0.12	−0.490 0.291	0.57
Creatinine [mg/dL]	−0.27	−0.564 0.09	0.14
TC [mg/dL]	0.25	−0.119 0.562	0.18
TG [mg/dL]	0.32	−0.044 0.611	0.08
LDL [mg/dL]	0.33	−0.035 0.616	0.08
HDL [mg/dL]	−0.16	−0.491 0.213	0.40
Wine			
Glucose [mg/dL]	−0.15	−0.513 0.262	0.48
Creatinine [mg/dL]	−0.21	−0.522 0.147	0.24
TC [mg/dL]	0.15	−0.224 0.483	0.43
TG [mg/dL]	−0.09	−0.439 0.276	0.62
LDL [mg/dL]	0.15	−0.225 0.482	0.44
HDL [mg/dL]	0.08	−0.286 0.430	0.66

(TC—total cholesterol, LDL—low-density lipoprotein cholesterol, HDL—high-density lipoprotein cholesterol, TG—triglycerides).

## Data Availability

The data that support the findings of this study are available from the corresponding author upon reasonable request.
